# Supplementary material for: Effectiveness of the Influence of Selected Essential Oils on the Growth of Parasitic Fusarium Isolated from Wheat Kernels from Central Europe
Source: Molecules. 2021 Oct 27;26(21):6488. doi: 10.3390/molecules26216488 (PMC8588391; doi:10.3390/molecules26216488)
Supplement: Supplementary file 1 [file molecules-26-06488-s001.zip › molecules-1422145-supplementary.pdf]

Supplementary materials

Table S1 Minimum of growth rate index of the analyzed oil at minimum concentration of the each considered isolates of Fusarium

Oil	Isolate	Concentration	N	Mean	SD.	Isolate	Concentration	N	Mean	SD.	Isolate	Concentration	N	Mean	SD.
grapefruit		2.000	4	41.275	0.596		2.000	4	14.133	4.414		2.000	4	24.813	2.275
cajeput		1.000	4	0.000	0.000		0.500	4	0.000	0.000		0.500	4	0.000	0.000
lemongrass		0.125	4	0.000	0.000		0.050	4	0.000	0.000		0.025	4	0.000	0.000
Litsea cubeba		0.125	4	0.000	0.000		0.050	4	0.000	0.000		0.025	4	0.000	0.000
thyme	FAPL	0.025	4	0.000	0.000	FCPL	0.025	4	0.000	0.000	FC1D	0.025	4	0.000	0.000
tea tree		0.500	4	0.000	0.000		0.500	4	0.000	0.000		0.500	4	0.000	0.000
verbena		0.125	4	0.000	0.000		0.125	4	0.000	0.000		0.050	4	0.000	0.000
Funaben T		0.125	4	0.000	0.000		0.125	4	0.000	0.000		0.125	4	0.000	0.000
Control			4	48.033	0.089			4	49.828	0.328			4	50.023	0.109
grapefruit		2.000	4	29.738	1.437		2.000	4	34.734	3.967		2.000	4	16.944	1.894
cajeput		0.500	4	0.000	0.000		0.500	3	0.000	0.000		0.500	4	0.000	0.000
lemongrass		0.050	4	0.000	0.000		0.050	4	0.000	0.000		0.050	4	0.000	0.000
Litsea cubeba		0.050	4	0.000	0.000		0.050	4	0.000	0.000		0.050	4	0.000	0.000
thyme	FC2D	0.025	4	0.000	0.000	FGPL	0.025	4	0.000	0.000	FG1D	0.025	4	0.000	0.000
tea tree		0.250	4	0.000	0.000		0.025	4	39.492	5.226		0.250	4	0.000	0.000
verbena		0.125	4	0.000	0.000		0.125	4	0.000	0.000		0.125	4	0.000	0.000
Funaben T		0.125	4	0.000	0.000		0.125	4	0.000	0.000		0.125	4	0.000	0.000
Control			4	49.184	0.131			4	49.863	0.187			4	51.722	0.082
grapefruit		2.000	4	18.016	0.491		2.000	4	29.233	1.252		2.000	4	20.547	0.172
cajeput		0.500	4	0.000	0.000		1.000	4	0.000	0.000		0.500	4	0.000	0.000
lemongrass		0.050	4	0.000	0.000		0.125	4	0.000	0.000		0.050	4	0.000	0.000
Litsea cubeba		0.050	4	0.000	0.000		0.125	4	0.000	0.000		0.050	4	0.000	0.000
thyme	FG2D	0.025	4	0.000	0.000	FOPL	0.125	4	0.000	0.000	FP0D	0.025	4	0.000	0.000
tea tree		0.250	4	0.000	0.000		0.500	4	0.000	0.000		0.250	4	0.000	0.000
verbena		0.050	4	0.000	0.000		0.125	4	0.000	0.000		0.125	4	0.000	0.000
Funaben T		0.125	4	0.000	0.000		0.125	4	0.000	0.000		0.125	4	0.000	0.000
Control			4	49.148	0.402			4	46.390	3.414			4	49.934	0.068
